# Supplementary material for: Holothurian Wall Hydrolysate Ameliorates Cyclophosphamide-Induced Immunocompromised Mice via Regulating Immune Response and Improving Gut Microbiota
Source: Int J Mol Sci. 2023 Aug 9;24(16):12583. doi: 10.3390/ijms241612583 (PMC10454611; doi:10.3390/ijms241612583)
Supplement: Supplementary file 1 [file ijms-24-12583-s001.zip › ijms-2526230-supplementary.pdf]

Table S1: The forward primer and reverse primer for qRT-PCR.

| Gene | Gene Accession Number | Primer Sequences 5'-3' | Product size (bp) |
|---|---|---|---|
| GAPDH | NM_001289726.1 | F: AGGTCGGTGTGAACGGATTTG<br>R: TGTAGACCATGTAGTTGAGGTC | 123 |
| TNF-α | NM_013693.3 | F: ATGTCTCAGCCTCTTCTCATTC<br>R: GCTTGTCACTCGAATTTTGAG | 179 |
| IL-6 | NM_031168.2 | F: CTCCCAACAGACCTGTCTATAC<br>R: ACTCCAGGTAGCTATGGTACTC | 258 |
| IL-1β | NM_008361.4 | F: TCGCAGCAGCACATCAACAAGAG<br>R: CTAATGGGAACGTCACACACCAGC | 209 |

Table S2: Summary of OTUs sequencing data attained.

| **Samples** | **OTUs Number** | **Sequences** | **Sample Size (bases)** | **OTUs Reads** |
|---|---|---|---|---|
| **NC1** | 530 | 113100 | 48110474 | 92903 |
| **NC2** | 396 | 102491 | 43723810 | 92756 |
| **NC3** | 315 | 83974 | 35713510 | 81194 |
| **M1** | 529 | 105648 | 44717689 | 75885 |
| **M2** | 495 | 89582 | 37974126 | 73128 |
| **M3** | 444 | 114530 | 48503613 | 105717 |
| **HWH-L1** | 636 | 99086 | 41894229 | 68860 |
| **HWH-L2** | 573 | 92149 | 39212706 | 74834 |
| **HWH-L3** | 548 | 117577 | 50019073 | 89014 |
| **HWH-H1** | 700 | 123618 | 52255791 | 95562 |
| **HWH-H2** | 555 | 98554 | 41610830 | 74018 |
| **HWH-H3** | 643 | 98316 | 41376776 | 73812 |
| **PC1** | 370 | 85293 | 35983737 | 83487 |
| **PC2** | 613 | 121490 | 51381017 | 91173 |
| **PC3** | 678 | 118720 | 50295278 | 89909 |
| **NH1** | 771 | 122252 | 51543743 | 84554 |
| **NH2** | 576 | 75637 | 31728517 | 57439 |
| **NH3** | 614 | 97212 | 41045291 | 66304 |

Table S3: Summary of alpha diversity indices estimation via 16S rRNA gene sequencing.

| **Samples** | **ACE** | **Chao 1** | **Shannon** | **Simpson** | **Coverage** |
|---|---|---|---|---|---|
| **NC1** | 583 | 571 | 3.52 | 0.0689 | 0.999 |
| **NC2** | 432 | 492 | 2.77 | 0.1913 | 0.999 |
| **NC3** | 344 | 345 | 2.75 | 0.17 | 0.999 |
| **M1** | 585 | 576 | 4.07 | 0.0317 | 0.998 |
| **M2** | 536 | 534 | 3.95 | 0.0374 | 0.999 |

| | | | | | |
|---|---|---|---|---|---|
| **M3** | 484 | 489 | 3.75 | 0.0414 | 0.999 |
| **HWH-L1** | 696 | 695 | 4.29 | 0.0355 | 0.998 |
| **HWH-L2** | 646 | 653 | 3.95 | 0.043 | 0.998 |
| **HWH-L3** | 612 | 596 | 3.88 | 0.0435 | 0.999 |
| **HWH-H1** | 763 | 753 | 4.27 | 0.035 | 0.999 |
| **HWH-H2** | 617 | 616 | 3.69 | 0.0906 | 0.998 |
| **HWH-H3** | 690 | 673 | 4.35 | 0.0356 | 0.998 |
| **PC1** | 380 | 378 | 4.07 | 0.0368 | 0.999 |
| **PC2** | 685 | 683 | 3.99 | 0.0372 | 0.998 |
| **PC3** | 734 | 721 | 3.94 | 0.0534 | 0.999 |
| **NH1** | 827 | 807 | 4.64 | 0.0205 | 0.998 |
| **NH2** | 646 | 648 | 4.37 | 0.0309 | 0.998 |
| **NH3** | 686 | 670 | 4.23 | 0.0287 | 0.998 |

Table S4: Percentage of bacterial phyla in different treatment groups.

| Groups | NC | M | HWH-L | HWH-H | PC | NH |
|---|---|---|---|---|---|---|
| ***Bacteroidetes*** | 18.3 | 69.25 | 63.10 | 45.82 | 68.19 | 65.48 |
| ***Firmicutes*** | 74.18 | 28.34 | 34.23 | 50.51 | 28.88 | 31.18 |
| ***Proteobacteria*** | 4.21 | 0.98 | 0.80 | 2.54 | 1.12 | 1.39 |
| ***Actinobacteria*** | 0.49 | 0.71 | 0.89 | 0.18 | 0.62 | 1.41 |
| ***Deferribacteres*** | 1.81 | 0.15 | 0.56 | 0.35 | 0.52 | 0.06 |
| ***Chloroflexi*** | 0 | 0 | 0 | 0 | 0 | 0 |
| ***Cyanobacteria*** | 0.01 | 0 | 0.08 | 0.15 | 0 | 0.01 |
| ***Gemmatimonadetes*** | 0 | 0 | 0 | 0 | 0 | 1.39 |
| ***Saccharibacteria*** | 0.15 | 0.09 | 0.06 | 0.06 | 0.13 | 0.18 |
| ***Tenericutes*** | 0.63 | 0.38 | 0.11 | 0.14 | 0.4 | 0.14 |
| ***Verrucomicrobia*** | 0.01 | 0 | 0.01 | 0.01 | 0.01 | 0 |

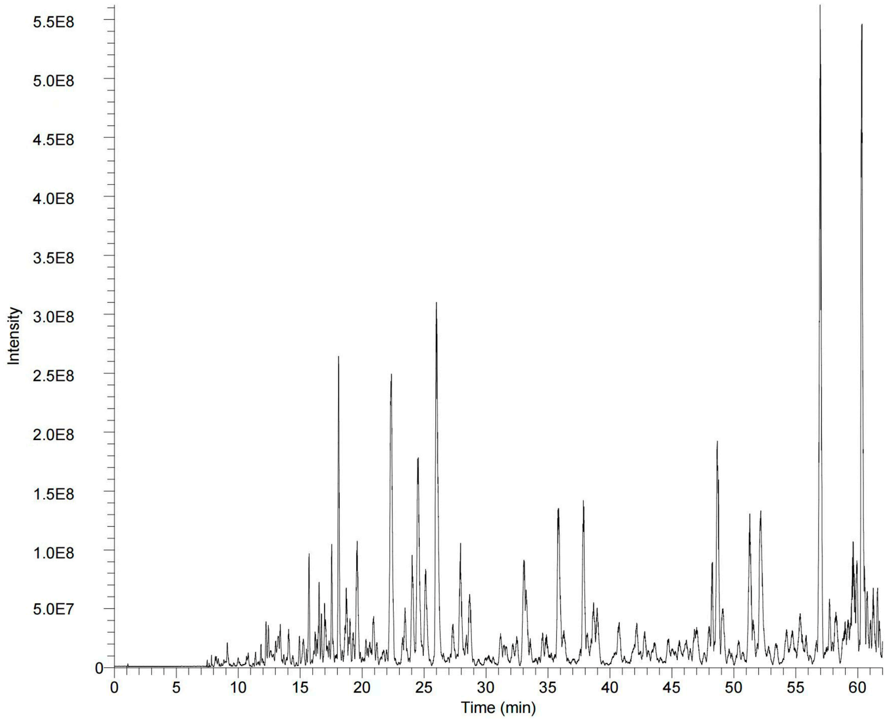


Figure S1. Total ion current diagram of HWH. Scanning range: 300-1400 m/z.
